# Effect of police enforcement and extreme social inequalities on violence and mental health among women who sell sex: findings from a cohort study in London, UK

**DOI:** 10.1136/sextrans-2021-055088

**Published:** 2021-10-26

**Authors:** Jocelyn Elmes, Rachel Stuart, Pippa Grenfell, Josephine Walker, Kathleen Hill, Paz Hernandez, Carolyn Henham, Sibongile Rutsito, MD Sarker, Sarah Creighton, Chrissy Browne, Marie-Claude Boily, Peter Vickerman, Lucy Platt

**Affiliations:** 1 Faculty of Public Health and Policy, London School of Hygiene & Tropical Medicine, London, UK; 2 University of Kent, Canterbury, UK; 3 University of Bristol, Bristol, UK; 4 Coventry University, Coventry, UK; 5 Homerton University Hospital, London, UK; 6 University of Leeds, Leeds, UK; 7 Barts Health NHS Trust, London, UK; 8 Infectious Diseases Epidemiology, Imperial College London, London, UK

**Keywords:** sex work, epidemiology, sexual health

## Abstract

**Objectives:**

To examine legal and social determinants of violence, anxiety/depression among sex workers.

**Methods:**

A participatory prospective cohort study among women (inclusive of transgender) ≥18 years, selling sex in the last 3 months in London between 2018 and 2019. We used logistic generalised estimating equation models to measure associations between structural factors on recent (6 months) violence from clients or others (local residents, strangers), depression/anxiety (Patient Health Questionnaire-4).

**Results:**

197 sex workers were recruited (96% cisgender-women; 46% street-based; 54% off-street) and 60% completed a follow-up questionnaire. Street-based sex workers experienced greater inequalities compared with off-street in relation to recent violence from clients (73% vs 36%); police (42% vs 7%); intimate partner violence (IPV) (56% vs 18%) and others (67% vs 17%), as well as homelessness (65% vs 7%) and recent law enforcement (87% vs 9%). Prevalence of any STI was 17.5% (17/97). For street-based sex workers, recent arrest was associated with violence from others (adjusted OR (aOR) 2.77; 95% CI 1.11 to 6.94) and displacement by police was associated with client violence (aOR 4.35; 95% CI 1.36 to 13.90). Financial difficulties were also associated with client violence (aOR 4.66; 95% CI 1.64 to 13.24). Disability (aOR 3.85; 95% CI 1.49 to 9.95) and client violence (aOR 2.55; 95% CI 1.10 to 5.91) were associated with anxiety/depression. For off-street sex workers, financial difficulties (aOR 3.66; 95% CI 1.64 to 8.18), unstable residency (aOR 3.19; 95% CI 1.36 to 7.49), IPV (aOR 3.77; 95% CI 1.30 to 11.00) and alcohol/drug use were associated with client violence (aOR 3.16; 95% CI 1.26 to 7.92), while always screening and refusing clients was protective (aOR 0.36; 95% CI 0.15 to 0.87). Disability (aOR 5.83; 95% CI 2.34 to 14.51), unmet mental health needs (aOR 3.08; 95% CI 1.15 to 8.23) and past eviction (aOR 3.99; 95% CI 1.23 to 12.92) were associated with anxiety/depression.

**Conclusions:**

Violence, anxiety/depression are linked to poverty, unstable housing and police enforcement. We need to modify laws to allow sex workers to work safely and increase availability of housing and mental health services.

## Background

Globally, sex workers can face high but varied levels of verbal, physical and sexual violence and interdependent poor mental, physical and sexual health. Between 19% and 44% of sex workers report physical workplace violence and 15%–61% intimate partner violence (IPV) in the previous year, and up to 18% report sexual or physical violence from police or acquaintances in the last year across diverse settings.^1 2^ Evidence shows interlinked and severe health consequences of violence against sex workers including physical and sexual injury, post-traumatic stress disorder (PTSD), depression and anxiety.^3 4^ Violence at any age, police arrest, work setting, gender/sexual minority identity and alcohol or drug use accounts for some of the high levels of PTSD (3%–64%), depression (24%–54%) and anxiety (5%–58%) experienced.^3 5^ Occupation-related stigma means sex workers are less likely to attend mental health services and may self-manage through drug or alcohol use.^6 7^ This has been associated with worse mental health, IPV and dependence on intimate partners for drugs, behaviours that can compound the adverse effects of poor physical and emotional health and are linked to decreased condom use elevating the risk of HIV/STI transmission.^3 8 9^

Evidence situates health inequities in relation to violence, mental and sexual health (including HIV infection) among sex workers in structural factors (eg, law, economy, stigma), workplace conditions (eg, settings, peer-led support, access to services) and interpersonal and individual behaviours (eg, sexual practices, drug use), intersecting with sex work criminalisation.^1 10 11^ Review evidence shows that police enforcement, mistreatment and discrimination disproportionately targets and stigmatises sex workers working on the street, those who use drugs, who are homeless, migrants or transgender and sex workers of colour.^11^ Inequalities exist between sex workers working indoors and those in street settings who tend to experience more violence and higher levels of depression and anxiety.^5 12 13^ Disparities have been linked to social exclusion including homelessness, regular crack or heroin use and repressive police practices.^5 13 14^ In turn, access to housing can be restricted by having a police record, leaving sex workers more exposed to violence and more visible to police.^1 15^

While selling sex is legal in the UK, many practices including soliciting in public and working together indoors under brothel-keeping laws are illegal. Sex workers also face enforcement under laws relating to drugs, immigration and public order.^16^ A recent call by an all-party parliamentary group to criminalise the purchase of sex throughout the UK with a view to ending demand for sex work was contested by sex workers, other civil society groups and academics for marginalising sex workers further.^17 18^ Alongside international bodies they argue in favour of decriminalising sex work to reduce harms against sex workers.^19 20^ Given the polarity of the debate and ongoing cuts to specialist services and broader social and health services across England and Wales,^21^ now is a critical time to generate evidence to inform policy and practice that protect sex workers’ health and well-being. This study quantifies the prevalence of violence, depression or anxiety and associations with law enforcement and other structural determinants using data from a prospective cohort of UK-based sex workers working in street and off-street settings.

## Methods

### Study design

The research was participatory. Academics and community co-researchers included people with lived experience of sex work or working with sex workers and provided expertise in sex work, service provision, epidemiology, sociology and criminology to develop the methods, gathering and analyse the data.

During May 2018–September 2019, sex workers of diverse genders across various settings in East London (Hackney, Newham and Tower Hamlets) were enrolled in a prospective open cohort completing a baseline and 6-month follow-up interview and offered voluntary chlamydia, gonorrhoea and HIV screening. To be eligible, participants had to have provided in-person sexual services in the previous 3 months in East London (later expanded to all London for off-street sex workers only) and be 18 years or older.

We mapped all sites in the areas (5 street and 103 online sites) for outreach and contact by phone or email. We used time-location sampling of street settings and targeted sampling of online profiles. We supplemented recruitment with convenience sampling (eg, in the National Health Service clinics, snowball sampling) and expanded baseline recruitment for street-based sex workers. The research team invited participants to self-complete a structured questionnaire on a tablet or online (Open Data Kit V.1.28.4) or, when requested, administered by the team. The questionnaire was available in English, Romanian, Polish, Brazilian-Portuguese with native speaking co-researchers interpreting. Three follow-up attempts of the original baseline sample were made by phone and email, supplemented with street outreach. Data were collected on demographic characteristics, organisation of sex work, health and social service use, mental and physical health, law enforcement, violence, sexual and substance use practices. Indicators were drawn from validated measures, other sex worker surveys and embedded in existing and emerging insights from a linked qualitative study.^2 22 23^

Self-administered chlamydia, gonorrhoea and HIV screening was offered in-person or by post. Alternatively, participants were asked if they consented to their last clinic test result to be linked to the research. In-person HIV screening used OraSure rapid oral tests; blood prick tests were used for postal screening and positive results confirmed by western blot analysis. Positive results were delivered by SC who arranged confirmatory testing. Negative results were delivered by text message or phone call within 72 hours by the project team.

All analyses focus on women (cis and trans) and are stratified by work setting, defined as the place sex workers met clients in the previous 6 months, in recognition of the disparities in police enforcement and the risk of violence by setting and gender.^5 12–14^

### Outcomes

For street-based sex workers, experience of violence and emotional health were analysed at baseline and follow-up: (1) recent (in the previous 6 months) physical or sexual violence from clients; (2) any recent emotional, physical or sexual violence from others (including local residents, strangers, drug dealers). For off-street sex workers, violence outcomes were limited to recent physical/sexual violence from clients (online supplemental table 1 summarises details of violence across perpetrators). We measured mental health over the last 2 weeks, through a validated composite measure (Patient Health Questionnaire-4) comprising two items from the Public Health Questionnaire focusing on depressed mood and loss of interest, and two items from Generalised Anxiety Disorder scale (feeling anxious, inability to control worrying). We took a cut-point of ≥ 6, which has been validated as a reliable brief measure of depression or anxiety.^24^

10.1136/sextrans-2021-055088.supp1 Supplementary data

### Covariables

Our key explanatory variable was direct exposure to law enforcement defined as arrest, client arrest, displacement from place of work, item-confiscation, referral to services by officers, imprisonment or immigration detention in previous 6 months or ever. A single combined variable included all items of law enforcement except imprisonment. Lifetime police violence is defined as ever experiencing verbal, physical or sexual assault by police. The indirect effect of police presence in a work area in the last 6 months was assessed by whether or not their presence reassured sex workers, deterred customers or meant sex workers had to rush negotiations with customers. We considered the following structural factors for their hypothesised relationships with violence and health: recent visit to a sex worker support project (ie, drug services, specialist sex worker services or sex worker-led groups); unmet mental health support need (wanting support for a mental health issue but not receiving any); difficulty meeting usual expenses; being in arrears; ever or recent (last 6 months) eviction and current (last 4 weeks) homelessness—sleeping rough or living in unstable accommodation.^23 25^ Aspects of sex work organisation included location of work, duration in sex work and safety strategies.

We adjusted for the following confounders for their association with policing and violence and poor health: age or duration in sex work, substance use (drug use in the last 4 weeks vs none, daily crack or heroin use vs not daily use/no crack or heroin use, alcohol or drug use (cut-point ≥5 on Alcohol Use Disorders Identification Test for Consumption, AUDIT-C, scale indicating increasing risk related to alcohol use or any drug use in last 4 weeks vs <5 on AUDIT-C and no drugs in last 4 weeks)) homelessness, number of hours worked per day or days per week (violence models only).^1 11 22^ We considered ethnicity, migration status (overseas national/refugee/asylum seeker/unknown vs UK nationality/permanent residence/indefinite leave to remain), education level, whether an intimate partner supplied drugs and existence of intimate partner in the previous 12 months as potential confounders.

### Statistical analyses

We examined univariable associations in separate models for each outcome using generalised estimating equation (GEE) logistic regression models with an exchangeable correlation matrix for all participants, taking into account the correlation of repeated observations. Variables significant at p<0.05 were included in separate multivariable models for each of violence and mental health outcomes. Variables in adjusted models with p<0.05 were considered statistically significant. Following the development of a multivariable model, we conducted a sensitivity analysis, exploring the association between outcomes and the key explanatory variable (direct and indirect law enforcement). We report baseline prevalence except where stated, and adjusted (aORs) and 95% CIs of the associations from GEE models incorporating baseline and follow-up. All analyses were conducted using R V.3.4.1.

## Results

**Figure 1:**
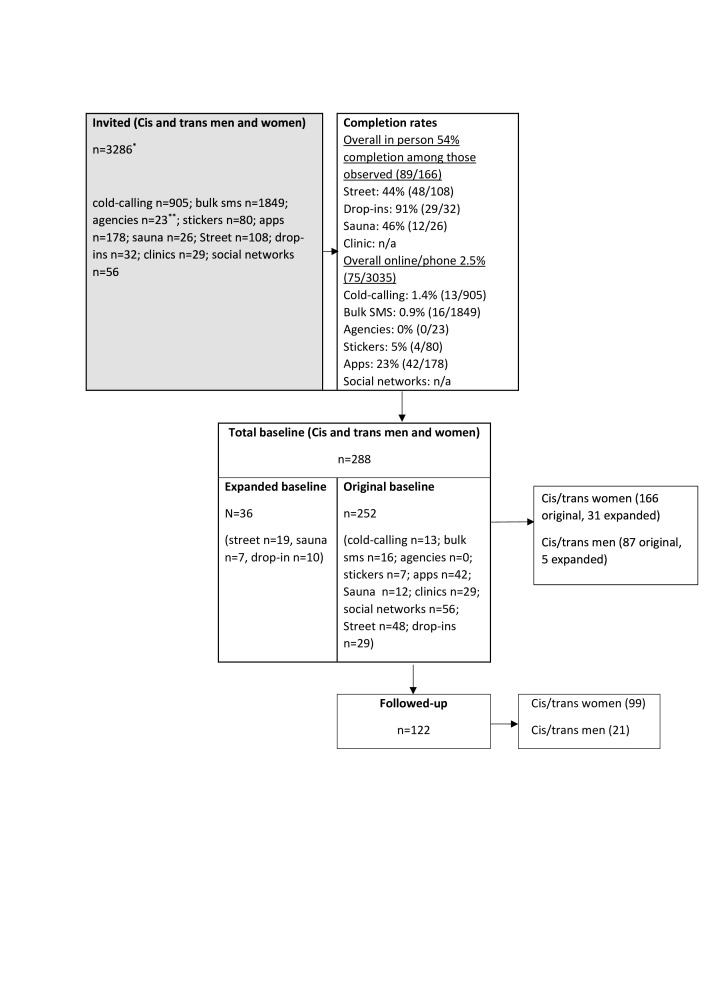
Flow diagram summarising recruitment and retention of participants. *As there are no data on sample frame for clinics and social networks, this denominator includes those interviewed at clinics/social network. †Twenty-three refers to the unique agencies or numbers that were contacted: seven people were connected via four sticker numbers that accepted visits (two unique flat shares). Therefore, the total participated is 252 since more than one individual participated from each number called. n//a, not available.

A total of 197 cis-female (190/197), trans-female and non-binary (7/197) sex workers completed baseline surveys (166 original baseline and 31 expanded baseline; overall in-person completion rate 54%; cold-calling/online completion rate 2.5%, figure 1), 99/166 of the original baseline sample were followed up (retention 60%) with a total of 296 observations over two visits; four people stopped sex work between surveys and were excluded from analyses. At baseline, just under half (90/197) found clients by working on the street (classified as street-based) and 54% (107/197) through massage parlours or online advertising in the previous 6 months (classified as off-street). Three-quarters (144/194) of the sample were white, 60% (117/195) were born in the UK, 27% (53/195) in other European countries and 13% (25/195) born outside Europe and 74% (144/195) had secure migration status. The median age was 34 years (IQR=28–42 years), the median age of sex work initiation was 23 years (IQR=18–28 years) and two-thirds (129/196) worked in East London. Demographics and working conditions differed substantially between work settings including always working alone, while safety strategies including screening and refusing clients (39%, 69/178) were comparable (table 1).

**Table 1:** Sample characteristics of the baseline and follow-up respondents by work setting

	Off-street		Street	
	Baseline (n=107)	Follow-up (n=60)	Baseline (n=90)	Follow-up (n=35)
**Demographic characteristics**				
**Age in years, median, (IQR**)	30.0 (25.0–37.5)	31.0 (26.0–37.3)	38.0 (32.3–45.0)	39.0 (33.5–47.5)
**Race/Ethnicity**				
People of colour (Asian, black, mixed/multiple ethnicities, other)	20/106 (19%)	7/58 (12%)	30/88 (34%)	16/34 (47%)
White	86/106 (81%)	51/58 (85%)	58/88 (63%)	18/34 (51%)
**Nationality status**				
Overseas national/refugee/asylum seeker/unknown	42/106 (40%)	16/58 (29%)	9/89 (10%)	3/35 (9%)
UK nationality/permanent residence/indefinite leave to remain	64/106 (60%)	41/58 (71%)	80/89 (90%)	32/35 (91%)
**Sexuality**				
Homosexual/Bisexual/Other terms/I do not use a term*	52/105 (50%)	33/55 (58%)	29/88 (32%)	11/34 (31%)
Heterosexual	53/105 (50%)	25/55 (42%)	59/88 (68%)	24/34 (69%)
**Sex working characteristics**				
Mainly work in East London (Hackney, Newham, Tower Hamlets)	45/106 (43%)	20/59 (34%)	84/90 (93%)	33/35 (94%)
Age at first sex work, median (IQR)	24.0 (20.0–30.0)	23.5 (19.0–31.0)	20.0 (17.0–25.8)	23.0 (18.0–27.5)
Years in sex work, median (IQR)	5.0 (2.0–9.0)	5.0 (3.0–11.0)	16.0 (7.0–23.0)	17.0 (11.5–22.0)
Number of days worked in last week, median (IQR)	3.0 (2.0–5.0)	2.0 (1.0–4.0)	5.0 (2.3–7.0)	3.0 (0.5–6.5)
Number of hours worked in last working day, median (IQR)	6.0 (3.0–11.0)	5.0 (3.0–7.3)	6.0 (2.0–10.0)	6.0 (2.5–8.0)
**Safety strategies used in last 6 months**				
Always work alone	53/95 (56%)	37/60 (62%)	59/83 (71%)	24/35 (69%)
Always screen and refuse clients	38/95 (40%)	22/60 (37%)	31/83 (37%)	9/35 (26%)
Always work where there is CCTV	25/85 (29%)	15/51 (29%)	22/80 (28%)	9/33 (27%)
**Health indicators**				
Depression and anxiety (PHQ-4 cut-point ≥6) in last 2 weeks	37/107 (35%)	30/60 (50%)	64/90 (71%)	24/35 (69%)
Ever attempted suicide	25/103 (24%)	13/57 (23%)	49/88 (56%)	22/31 (71%) 10
Physical or mental impairment limiting daily activities in last 6 months	32/103 (31%)	25/60 (42%)	50/88 (57%)	20/35 (56%)
Alcohol use (AUDIT-C cut-point ≥5)	40/105 (38%)	26/60 (43%)	31/89 (35%)	11/35 (32%)
Current drug use (used recreational drugs in the last 4 weeks)	43/104 (41%)	29/58 (50%)	82/90 (91%)	33/33 (100%)
Daily crack or heroin use	<5/107	<5/60	66/90 (73%)	25/35 (71%)
Any STI among those testing	9/46 (20%)	<5/6	8/51 (17%)	<5/11
Chlamydia among those testing	8/46 (17%)	<5/6	<5/51	<5/11
Gonorrhoea among those testing	<5/45	0/6	7/51 (14%)	<5/11
**Violence by perpetrator in last 6 months**				
Physical/Sexual violence from clients†	38/105 (36%)	18/60 (30%)	65/89 (73%)	23/34 (68%)
Any violence from intimate partners‡‡	19/105 (18%)	14/59 (24%)	49/88 (56%)	14/34 (41%)
Any violence from other perpetrators‡	18/104 (17%)	16/60 (27%)	58/87 (67%)	20/33 (61%)
Any police violence§	7/105 (7%)	<5/60	37/89 (42%)	12/34 (35%)
Reported violence to police in last 6 months (n=any recent violence)	7/69 (10%)	<5/43	14/80 (18%)	6/29 (21%)
**Historical police enforcement**				
Ever arrested	24/103 (23%)	18/60 (30%)	80/88 (90%)	31/34 (91%)
Ever been to prison	6/105 (6%)	7/59 (12%)	61/88 (69%)	23/32 (72%)
Ever detained by immigration officers	8/105 (8%)	<5/60	5/88 (6%)	<5/33
**Recent police enforcement variables (last 6 months**)				
Displaced from working premises/area¶	7/106 (7%)	<5/58	68/88 (77%)	22/35 (63%)
Items confiscated by police	0 (0%)	<5/60	33/89 (37%)	10/34 (29%)
Client arrested	<5/106	0/59 (0%)	28/87 (32%)	8/31 (26%)
Been referred to social or health services**	<5/104	<5/59	14/86 (16%)	9/35 (26%)
Stopped, interviewed or detained by immigration services/officers in the UK	<5/105	0/60 (0%)	<5/88	<5/32
Arrested, detained or charged for any reason by UK police	<5/103	<5/60	43/87 (48%)	14/33 (42%)
Arrested/cautioned/received warning/notice (sanctioned)	6/106 (6%)	<5/60	62/89 (70%)	19/35 (54%)
Experienced any law enforcement††	10/107 (9%)	7/59 (12%)	78/90 (87%)	26/35 (74%)
**Police presence in the area affected work in last 6 months through:**	6/101 (6%)	<5/59	67/89 (74%)	24/33 (71%)
Deterred clients	<5/101	<5/59	50/89 (56%)	21/33 (64%)
Meant I had to rush negotiations with clients	<5/101	0/59 (0%)	45/89 (51%)	17/33 (52%)
Moved to new location/provided services away from main roads/in secluded places	<5/101	<5/59	42/89 (47%)	12/33 (36%)
**Other structural determinants**				
Homeless§§ in last 4 weeks	7/106 (7%)	10/60 (17%)	58/89 (65%)	19/34 (56%)
Ever evicted	15/104 (14%)	13/60 (22%)	45/87 (52%)	19/33 (58%)
Child taken into care (ever)	<5/104	5/58 (9%)	30/85 (35%)	8/31 (26%)
In arrears (at time of survey)	36/98 (37%)	29/59 (49%)	39/68 (57%)	14/27 (52%)
Difficulty paying usual expenses (at time of survey)	57/107 (53%)	33/60 (55%)	66/90 (73%)	24/35 (69%)
**Healthcare access in the last 6 months**				
Visited a sex worker support project	36/107 (34%)	22/60 (37%)	43/90 (48%)	21/35 (60%)
Receiving support for a mental health problem	32/103 (31%)	22/58 (38%)	37/87 (43%)	14/34 (41%)
Unmet mental health need (wanted treatment, but had not received it)	29/101 (29%)	20/59 (34%)	46/87 (53%)	19/35 (54%)

<5 refers to categories with fewer than five individuals that cannot be combined. Denominators do not always sum to total due to missing data.

*Numbers in different categories across follow-up were too small to present separately without disclosure.

†Combines physical violence, hostage taking, removal of condom without consent, sexual assault, forced sexually degrading acts, rape.

‡Combines verbal abuse, physical violence and rape.

§Combines verbal abuse and intimidation from police, and police damage to property, physical violence, sexual assault from police, police demanding sex in exchange for no arrest or to avoid trouble, and rape by police officers.

¶Combines displacement from area where working and raided or evicted from living or working premises.

**Referral to services by police includes mandatory service referrals to avoid arrest (court diversion schemes) and is criticised as a policy of enforced welfare.^32^

††Combines displacement, sanction (issued with caution, notice, warning by police), confiscation of valuables, drug paraphernalia or condoms, referral to services, stopped/detained/interviewed by immigration officers, arrest, client arrest.

‡‡Combines abusive language, belittling or humiliating, scaring/intimidation, stalking, outing/threats to out, theft, physical violence, hostage taking, removal of condom without consent, sexual assault, forced sexually.

§§Homeless is defined as sleeping rough or living in unstable accommodation (eg, parent’s or friend’s home, sheltered or homeless accommodation).

AUDIT-C, Alcohol Use Disorders Identification Test for Consumption; PHQ-4, Patient Health Questionnaire-4.

### Violence, emotional health and STIs

Overall, 53% (103/194) experienced recent physical or sexual violence from clients, 40% (76/191) any violence from others, 35% (68/193) any IPV and 23% (44/194) any violence from police. Just 14% (21/149) mentioned reporting recent violence to police, citing lack of confidence of being treated fairly or taken seriously as the most common deterrent. Overall, 51% (101/197) of the sample had symptoms of anxiety and depression. Violence across all perpetrators and poor mental or physical health were more prevalent among women working in outdoor settings than women working off-street.

Just under half the sample consented to testing or linking (97/197) among whom just 53 (27% overall) consented to HIV testing; uptake for postal self-testing was low (6/61). Among those testing, prevalence of any STI was 17.5% overall (17/97), with little difference by work setting (table 1). Overall prevalence of chlamydia and gonorrhoea among those testing was 11.3% and 10.4%, respectively. Chlamydia was higher among those working off-street but gonorrhoea higher among those working in street settings (table 1). No HIV infections were detected among those tested.

### Structural factors

Lifetime and recent law enforcement was ubiquitously high but disproportionately experienced by those working outdoors, 87% (78/90) of whom had experienced recent police enforcement. Indirect policing affected street-based sex work by deterring clients (56%, 50/89) and resulting in rushing negotiations with clients (51%, 45/89); just 3% found police presence reassuring or made working safer.

A higher proportion of those working outdoors were homeless (65% (58/89) vs 7% (7/106)) than those working off-street and experienced financial difficulty (73% (66/90) vs 53% (57/107)). Three-quarters (66/90) of those working outdoors use crack or heroin daily whereas 59% (61/104) of those working off-street reported no drug use in the last month. Recent attendance at a sex worker support project was higher among those working outdoors (48%) compared with off-street (34%). Just over a third of the sample were receiving support for a mental health problem, but a further 40% stated they wanted but had not received support in the last 6 months; need was greatest among those working outdoors (53% vs 29%).

### Correlates of violence and emotional health among street-based sex workers

Sex workers who had been recently displaced by police (aOR 4.35; 95% CI 1.36 to 13.90), experienced financial difficulties (aOR 4.66; 95% CI 1.64 to 13.24) and had accessed a sex worker support project (aOR 3.54; 95% CI 1.27 to 9.89) were more likely to have experienced physical or sexual violence from clients adjusting for duration in sex work, daily crack or heroin use and homelessness (table 2). Sex workers who have been recently arrested for any reason (aOR 2.77; 95% CI 1.11 to 6.94) and experienced recent IPV (aOR 4.00; 95% CI 1.64 to 9.72) were also more likely to experience violence from other perpetrators. In sensitivity analyses, recent referral to services by police was associated with violence from others, while recent arrest of clients and past experience of police violence were associated with client violence.

**Table 2:** Unadjusted and adjusted associations with recent violence across a range of perpetrators reported by street-based sex workers

	Physical or sexual violence from clients		Any violence from others		Depression/Anxiety (cut-point >5)	
	Unadjusted	Adjusted	Unadjusted	Adjusted	Unadjusted	Adjusted
**Individual/Partnership-level factors**	**GEE OR (95% CI**)	**GEE aOR (95% CI**)	**GEE OR (95% CI**)	**GEE aOR (95% CI**)	**GEE OR (95% CI**)	**GEE aOR (95% CI**)
People of colour (vs white)	0.62 (0.26 to 1.49)	–	1.02 (0.45 to 2.33)	–	0.60 (0.25 to 1.48)	–
Bisexual, homosexual, any other term (vs heterosexual)	1.73 (0.67 to 4.49)	–	1.20 (0.49 to 2.97)	–	2.82 (1.14 to 6.96)	3.55 (1.30 to 9.71)
Unstable residency status (vs permanent/UK resident)††	0.70 (0.17 to 2.92)	–	0.68 (0.16 to 2.79)	–	0.77 (0.18 to 3.33)	–
Primary or secondary education (vs further education)	1.17 (0.54 to 2.50)	–	0.86 (0.40 to 1.85)	–	1.30 (0.53 to 3.16)	–
Partner supplies drugs	1.12 (0.48 to 2.60)	–	2.11 (0.94 to 4.72)			
Daily crack or heroin use	1.56 (0.66 to 3.69)	1.18 (0.41 to 3.43)	2.18 (0.91 to 5.21)	2.78 (0.91 to 8.55)	1.40 (0.51 to 3.83)	0.68 (0.22 to 2.06)
Any intimate partner violence in last 6 months	2.12 (0.91 to 4.96)	–	2.87 (1.35 to 6.11)	4.00 (1.64 to 9.72)	–	–
Physical/Sexual violence from clients in last 6 months	Not included		2.80 (1.16 to 6.80)	–	2.30 (1.18 to 4.48)	2.55 (1.10 to 5.91)
Limited/Severely limited by disability (vs no disability)	Not included*		Not included*		3.21 (1.52 to 6.78)	3.85 (1.49 to 9.95)
**Workplace factors**						
Provides services in vehicles	2.93 (1.19 to 7.20)	–	Not included*		Not included*	
Duration in sex work ≥15 years†	0.61 (0.26 to 1.41)	0.43 (0.16 to 1.15)	0.89 (0.42 to 1.88)	1.02 (0.41 to 2.53)	0.77 (0.37 to 1.62)	0.73 (0.28 to 1.88)
Number of days worked in the last week >5	4.55 (1.89 to 10.97)	3.04 (1.16 to 7.96)	1.23 (0.57 to 2.67)	–	Not included*	
Always work alone	0.93 (0.35 to 2.42)	–	Not included*		0.87 (0.41 to 1.85)	–
Always work in areas where there is CCTV	0.99 (0.36 to 2.77)	–	Not included*		Not included*	
Always screen and refuse clients	1.12 (0.51 to 2.42)	–	Not included*		Not included*	
Always work in well-lit areas	0.89 (0.34 to 2.35)	–	Not included*		Not included*	
**Structural variables— law enforcement‡**						
Displaced from area by police in last 6 months	5.24 (2.18 to 12.6)	4.35 (1.36 to 13.90)				
Arrest for any reason in last 6 months			2.72 (1.18 to 6.27)	2.77 (1.11 to 6.94)		
**Denied access to criminal justice§**					2.91 (1.23 to 6.86)	1.95 (0.74 to 5.15)
**Structural variables—other**						
Homeless in the last 4 weeks‡‡	2.75 (1.32 to 5.71)	1.87 (0.74 to 4.74)	1.48 (0.70 to 3.14)	0.88 (0.36 to 2.14)	2.47 (1.05 to 5.80) 0	2.19 (0.81 to 5.88)
Accessed sex worker support project in last 6 months	2.55 (1.13 to 5.72)	3.54 (1.27 to 9.89)	1.08 (0.50 to 2.34)	–	0.83 (0.42 to 1.64)	–
Unmet mental health support need in last 6 months	1.65 (0.69 to 3.91)	–	2.20 (1.03 to 4.68)	–	1.11 (0.62 to 2.00)	–
Find it difficult to pay usual expenses (at time of survey)	2.86 (1.32 to 6.23)	4.66 (1.64 to 13.24)	0.86 (0.39 to 1.87)	–	1.80 (0.88 to 3.70)	–
**Sensitivity analyses¶**						
Ever experienced violence from police	3.24 (1.60 to 6.57)	3.77 (1.46 to 9.73)			1.67 (0.83 to 3.33)	–
Client arrested in last 6 months	2.63 (0.99 to 6.96)	3.61 (1.11 to 11.78)				
Referred to services in last 6 months			2.05 (0.73 to 5.73)	2.91 (1.01 to 8.37)		
Police presence deters punters**					2.65 (1.27 to 5.51)	2.32 (1.04 to 5.16)
Police presence causes rushed negotiations**					3.00 (1.41 to 6.37)	4.15 (1.84 to 9.39)

– denotes variables excluded in adjusted models not significant in adjusted models at p<0.05 and not a priori confounders.

*Not considered a potential confounder.

†Age or duration of sex work were selected as a priori confounders on the basis of quasi-AIC (QIC) evaluations.

‡Multivariable models adjust for one policing variable.

§Denied access to justice was defined as not reporting an episode of violence to police or reported violent episode but the police either arrested the sex worker or failed to take the report seriously.

¶Sensitivity analyses tested all policing variables in separate models adjusted for the variables shown excluding other policing variables. Only those with significant effect sizes (p<0.05) in adjusted analyses are displayed.

**Response to two-part question about how police activity in the last 6 months in the area affects work.

††Unstable defined as overseas national/asylum seeker/unknown or illegal migration status (vs permanent residency/UK national/settled status).

‡‡Homelessness defined as sleeping rough or living in unstable accommodation (eg, parent’s or friend’s home, sheltered or homeless accommodation).

GEE, generalised estimating equation.

Sex workers who had experienced recent sexual or physical violence from clients (aOR 2.55; 95% CI 1.10 to 5.91) and had a limiting disability (aOR 3.85; 95% CI 1.49 to 9.95) were more likely to have symptoms consistent with depression or anxiety (table 2). In sensitivity analyses, police presence resulting in rushed negotiations and deterring customers were associated with depression or anxiety.

### Correlates of violence and emotional health among off-street sex workers

In multivariable models (table 3), sex workers having difficulties paying usual expenses (aOR 3.66; 95% CI 1.64 to 8.18), lifetime experience of IPV (aOR 3.77; 95% CI 1.30 to 11.00), who had unstable residency status (aOR 3.19; 95% CI 1.36 to 7.49) or who reported alcohol or drug use (aOR 3.16; 95% CI 1.26 to 7.92) were more likely to experience physical or sexual violence from clients, while always screening and refusing clients was protective (aOR 0.36; 95% CI 0.15 to 0.87). Sex workers who had ever had items confiscated (aOR 4.59; 95% CI 0.99 to 21.20), been evicted (aOR 3.99; 95% CI 1.23 to 12.92), who wanted but had not received support for a mental health problem (aOR 3.08; 95% CI 1.15 to 8.23) and who were limited by disability (aOR 5.83; 95% CI 2.34 to 14.51) were more likely to be depressed or anxious. In sensitivity analyses, sex workers who had ever been detained by immigration officers were also more likely to have depression or anxiety (table 3).

**Table 3:** Unadjusted and adjusted associations with physical/sexual violence from clients, depression and anxiety among off-street sex workers

	Physical or sexual violence from clients in last 6 months		Depression or anxiety (cut-point >5)	
	Unadjusted	Adjusted	Unadjusted	Adjusted
**Individual-level factors**	**GEE OR (95% CI**)	**GEE aOR (95% CI**)	**GEE OR (95% CI**)	**GEE aOR (95% CI**)
Age ≥30 years*	0.40 (0.19 to 0.83)	0.48 (0.21 to 1.13)	0.36 (0.18 to 0.72)	0.33 (0.13 to 0.86)
People of colour (vs white)	1.00 (0.39 to 2.60)	–	0.65 (0.28 to 1.51)	–
Bisexual, homosexual, any other term (vs heterosexual)	1.29 (0.64 to 2.57)	–	2.03 (1.03 to 4.01)	–
Unstable residency status (vs permanent residency/UK national)†	1.67 (0.84 to 3.31)	3.19 (1.36 to 7.49)	0.41 (0.20 to 0.85)	–
Primary or secondary education only (vs further education)	1.11 (0.56 to 2.18)	–	0.89 (0.49 to 1.61)	–
Alcohol or drug use‡	2.85 (1.36 to 5.94)	3.16 (1.26 to 7.92)	2.19 (1.13 to 4.24)	2.19 (0.91 to 5.28)
Any intimate partner violence ever	3.70 (1.61 to 8.47)	3.77 (1.30 to 11.00)	2.92 (1.35 to 6.31)	–
Physical/Sexual violence from clients in last 6 months	Not included	–	2.12 (1.18 to 3.80)	–
Limited/Severely limited by disability (vs no disability)	Not included§	–	6.86 (3.28 to 14.37)	5.83 (2.34 to 14.51)
**Workplace factors**				
Duration in sex work ≥5 years*	0.74 (0.38 to 1.45)	Not included*		
Hours worked per day >8 hours	1.98 (1.08 to 3.63)	–	Not included§	–
Always work alone	0.79 (0.38 to 1.60)	–	1.12 (0.62 to 2.01)	–
Always work with CCTV	0.53 (0.26 to 1.10)	–	Not included§	–
Always screen and refuse clients	0.36 (0.17 to 0.76)	0.36 (0.15 to 0.87)	Not included§§	–
Always have security/maid	0.44 (0.16 to 1.21)	–	Not included§	–
Always work check customer’s number with a safety service	1.31 (0.56 to 3.03)	–	Not included§	–
**Structural variables—law enforcement¶**				
Ever arrested by police	0.96 (0.46 to 2.01)	0.53 (0.19 to 1.45)	2.36 (1.29 to 4.32)	–
Ever had items confiscated	–	–	7.35 (2.02 to 26.81)	4.59 (0.99 to 21.20)
**Structural variables—other**				
Homeless in the last 4 weeks††	1.75 (0.71 to 4.32)	–	2.53 (1.00 to 6.39)	–
Accessed sex worker support project in last 6 months	0.84 (0.40 to 1.84)	–	1.23 (0.69 to 2.19)	–
Unmet mental health support need in last 6 months	1.66 (0.81 to 3.39)	–	5.99 (2.61 to 13.72)	3.08 (1.15 to 8.23)
In arrears (at time of survey)	1.38 (0.68 to 2.79)	–	2.12 (1.23 to 3.67)	–
Difficulty paying usual expenses	3.14 (1.50 to 6.58)	3.66 (1.64 to 8.18)	2.52 (1.29 to 4.93)	–
Ever evicted	Not included§		3.66 (1.48 to 9.05)	3.99 (1.23 to 12.92)
Sensitivity analyses**				
Ever detained by immigration officers			2.22 (0.75 to 6.60)	5.06 (1.43 to 17.93)

– denotes variables excluded in adjusted models not significant in adjusted models at p<0.05 and not a priori confounders.

*Age or duration of sex work were selected as a priori confounders on the basis of quasi-AIC (QIC) evaluations.

†Unstable defined as overseas national/asylum seeker/unknown or illegal migration status (vs permanent residency/UK national/settled status).

‡Alcohol use or drug use defined as (AUDIT-C score of 5 or more indicating increasing risk) or used drugs in the last month.

§Not considered to be a potential confounder.

¶Multivariable models adjust for one policing variable.

**Sensitivity analyses tested all policing variables in separate models adjusted for the variables shown excluding other policing variables. Only those with significant effect sizes (p<0.05) in adjusted analyses are presented.

††Homelessness defined as sleeping rough or living in unstable accommodation (eg, parent’s or friend’s home, sheltered or homeless accommodation).

## Discussion

We find very high levels of poor mental health, STIs, alongside widespread violence from clients, intimate partners and other members of the community among a cohort of largely cis-female UK-based sex workers. This is important new evidence demonstrating that sex workers’ safety, and their mental health, are impacted by law enforcement and extreme precarity. Sex workers working on the street are particularly targeted by police through displacement from work areas (77%), being cautioned or arrested (70%) or experiencing police violence (42%). Recent arrest or displacement from a work location were associated with increased risk of violence from clients and others. While experience of recent policing was less frequent among those working off-street, lifetime experience of being detained by immigration officers was linked to depression or anxiety. Financial difficulties were linked to client violence among all sex workers and unstable housing (in the form of past eviction) to anxiety or depression among off-street sex workers.

Violence from clients, depression or anxiety were approximately twice as high, IPV and violence from others were 3–4 times higher among street-based sex workers than those working off-street, disparities that are in line with other studies.^12 13^ Our findings concur with other evidence demonstrating that enforcement tactics (including against clients) increase vulnerability to physical and sexual violence from clients and other perpetrators.^2 22 26^ We found that screening and refusing clients was protective against client violence for those working off-street but no evaluated safety strategies were protective for those working in street settings. Protective effects may be attenuated by continued police presence on the streets which deterred customers, meaning sex workers had to rush negotiations and move their place of work, which contributed to increased odds of anxiety and depression. This practice runs contrary to current National Police Chief Council (NPCC) guidance that states displacement should be avoided for welfare and safety of sex workers and advocates a victim-centred approach to build trust acknowledging sex workers’ vulnerabilities.^27^ However, just 14% of our sample had reported violent crimes to the police suggesting low levels of trust. By contrast, in New Zealand, decriminalisation has meant improved relationships between sex workers and police and increased safety among sex workers.^11^ We found a similar prevalence of STI to a comparable study among off-street sex workers conducted in 2011.^28^ The high sample positivity and failure to reduce STI prevalence may in part be due to cuts to sexual health services over the last decade, although the low uptake of STI testing particularly among off-street sex workers means these results need to be interpreted cautiously.

Separating analyses of violence by perpetrator provides a more complete picture, highlighting the role of police and community members and the inter-relationships between violence across perpetrators.^2 22^ For example, IPV was an important correlate of violence from others among street-based sex workers and with clients among off-street, as evidenced elsewhere.^29^ While the mechanisms through which IPV may influence interactions with clients or others are not clear, we found an independent association with drug/alcohol use and client violence alongside IPV, indicating partners may exercise control over drug use and selection of clients as shown elsewhere.^8 22^ Contrary to other evidence, current homelessness was not linked to recent violence in adjusted analyses from any perpetrator among street-based sex workers.^22^ Failure to detect similar associations might be due to the broader definition of homelessness used (encompassing unstable accommodation and not solely sleeping on the street).

Police enforcement, migration status, unstable housing and financial difficulties were clearly linked to increased risk of violence, depression or anxiety.^4 5^ Funding for multilevel interventions that can address these complex and inter-related health needs is imperative. Services that place sex workers’ needs centrally is critical including in relation to drug treatment and criminal justice advocacy, rather than prioritising exiting which can leave many sex workers without support.^23 30^ Structural changes that increase social housing supply improve the welfare system and include those without recourse to public funds needs to occur simultaneously. This would both help address the conditions that perpetuate violence experienced by sex workers in all aspects of their lives and create an environment in which other employment options become financially viable.^31^

Research to monitor the effect of changes to sex work laws, housing and the economy on sex workers’ health and welfare, as well as the intergenerational effects of criminalisation, discrimination and poverty is needed. Given the prospect of economic austerity in response to the effects of the SARS-COV-2 pandemic, this is imperative. Realist informed trials of different policing models, designed and implemented in partnership with sex worker-led organisations, would provide more rigorous evidence on effective models to protect sex workers as well as measure interaction with structural determinants. Finally, research into the barriers to accessing mental and other health services is critical to understanding why mental health needs observed here and elsewhere are woefully underserved.

### Limitations

Although the observational design of the study precludes determining causality between exposures and outcomes, longitudinal analyses using GEE models accounting for repeated responses allow for estimation of the associations over time, although limited by the fairly low follow-up rate. Despite extensive attempts to recruit a probabilistic sample, recruitment had to be boosted with convenience methods, limiting inferences we can make on the representativeness of findings to other sex workers outside of London. The small numbers of off-street sex workers reporting enforcement likely reflects difficulties in recruiting people working in shared premises who had experienced raids or displacement by police and impedes our understanding of how they are policed. This may also have affected power to detect associations between law enforcement and client violence in off-street sex workers. The association between immigration enforcement and anxiety/depression shows how non-sex work laws are used to enforce against sex work and the adverse health consequences of this, although with some uncertainty as a result of lack of access to immigration detention centres as well as failures to resume contact with individuals who were deported. We also had difficulty in recruiting the small group of migrant women (predominantly Romanian) working in street-based settings, precluding any analysis of the effect of enforcement on migrant street-based sex workers. Finally, uptake of testing was low (10%) among those surveyed online and limits inferences about prevalence of STI among off-street sex workers.

## Conclusion

Experiences of violence, frequent drug use, homelessness and poor mental health were comparable or higher than 10–20 years ago, highlighting the ongoing cycle of poverty and social exclusion experienced by sex workers.^12 13 28^ This has occurred despite NPCC guidance and amendments to laws governing sex work designed to shift burden of policing from sex worker to client. Our findings underline the urgency for social and legal policy reform ceasing enforcement against sex workers, their clients, tackling stigma and decriminalising sex work to allow sex workers to work safely. In the short term, increased provision of housing must be prioritised, alongside strengthening of existing specialist sex worker and support services to address violence, drug treatment needs, sexual and mental health. Failure to do so risks perpetuating and exacerbating poor health and violence against sex workers.

**Key messages**

- Findings show that violence against sex workers alongside poor mental health is strongly linked to precarity in the form of unstable housing, migration and financial difficulties and compounded by police enforcement practices and criminalisation, particularly for those working on the street.
- Violence from clients, depression and anxiety were approximately twice as high, intimate partner violence was 3 times as high and violence from other perpetrators was 4 times as high among street-based sex workers than those working off-street.
- The extent of violence experienced by sex workers from intimate partners, other members of the community and police is comparable with violence from clients highlighting the importance of expanding interventions to address diverse perpetrators and the underlying drivers of violence.
- Findings support the removal of criminal sanctions that target sex workers and their clients to improve safety and mental health and point to the need to improve the conditions in which sex workers live and work including increasing access to welfare, housing and health and social services.

10.1136/sextrans-2021-055088.supp2 Supplementary data

## Data Availability

Data are available on reasonable request. Data will be made available on reasonable request.
