# Supplementary material for: Effect of police enforcement and extreme social inequalities on violence and mental health among women who sell sex: findings from a cohort study in London, UK
Source: Sex Transm Infect. 2021 Oct 26;98(5):323–31. doi: 10.1136/sextrans-2021-055088 (PMC9340007; doi:10.1136/sextrans-2021-055088)
Supplement: Supplementary data [file sextrans-2021-055088supp001.pdf]

Supplement Table 1: Definitions of violence

Type of violence used in analysis	Perpetrator type of violence applies to			
**Emotional violence**	**Clients[1]**	**Intimate partners[2]**	**Others[3]**	**Police**
Perpetrator belittled or humiliated you or used abusive or insulting language towards you such as calling you inappropriate names or making racist remarks.		✓	✓	✓
Perpetrator done things to scare or intimidate you on purpose or threatened to hurt you or someone you care about.		✓	✓	
Perpetrator made or attempted repeated unwanted contact online, by phone or in person including following you (stalked).		✓		
Perpetrator threatened to tell others (e.g. landlord, neighbours, police, immigration, friends, family, publish online) that you do sex work ("out" you).		✓		
Perpetrator stolen or attempted to steal from you (money or possessions or drugs taken) or refused to pay.		✓		
Perpetrator done things to scare or intimidate you or made sexual comments to you or inappropriately commented on your appearance				✓
Perpetrator damaged personal property				✓
**Physical violence**	**Clients[1]**	**Intimate partners[2]**	**Others[3]**	**Police**
Perpetrator physically abused you (pushed, shoved, slapped, kicked, punched, choked, dragged, burned you, used a weapon against you, thrown something at you, beaten you up).	✓	✓	✓	✓
Perpetrator held or taken you against your will, even for a short time (taken hostage or kidnapped or abducted).	✓	✓		
**Sexual violence**	**Clients[1]**	**Intimate partners[2]**	**Others[3]**	**Police**
Perpetrator pressured you to have sex without a condom against your will or removed a condom without consent.	✓	✓		

Perpetrator touched or grabbed you sexually against your will (grope) or attempted to get sex through force/threat (sex includes oral, vaginal or anal sex).	✓	✓		✓
Perpetrator forced you to do something sexual that you found degrading or humiliating	✓	✓		
Perpetrator forced you to have sex when you did not want to (sex includes oral, vaginal or anal sex).	✓	✓	✓	✓
Perpetrator inappropriately touched you (groped)				✓
Perpetrator offered accepted sex, money or other goods from you in exchange for no arrest, to avoid hassle or to avoid trouble				✓

[1]Includes clients (paying customers) or people posing as clients. Although participants were asked about emotional violence from clients, only physical or sexual violence was considered in analysis due to ubiquity of emotional violence among sex workers working on the street; [2]Intimate partners includes spouse, boyfriend, girlfriend or other sexual partner who is not a client; [3]Others includes residents (where live/work), strangers, family members/relatives, other sex workers, drug dealers, co-worker/boss (not in sex work), receptionist/cleaner, security/driver, manager, pimp, police
