# Supplementary material for: Effect of police enforcement and extreme social inequalities on violence and mental health among women who sell sex: findings from a cohort study in London, UK
Source: Sex Transm Infect. 2021 Oct 26;98(5):323–31. doi: 10.1136/sextrans-2021-055088 (PMC9340007; doi:10.1136/sextrans-2021-055088)
Supplement: Supplementary data [file sextrans-2021-055088supp002.pdf]

Supplement

The effect of police enforcement and extreme social inequalities on violence and mental health among women who sell sex: findings from a cohort study in London, UK

Detailed methods

Sampling

Achieving a probabilistic sample through time-location sampling or systematic random sampling requires a sampling frame where the population size of the sex worker population must be estimated. We mapped all the known locations where sex workers provide services to create three sampling frames for sex workers based on where they work: 1) individuals working from flats or as independent escorts who advertise on their own websites or advertising on platforms; 2) sex workers working in saunas or massage parlours; 3) sex workers working on the street. In brief, online mapping involved systematic google searches for geographic tags and key search terms for promoting sexual services that were co-developed with sex workers. The first 100 results for each search was entered into a spreadsheet, the list was deduplicated and details on 103 unique London-based sites were identified with an estimated 32000 profiles (estimation based on numbers of photos/adverts placed on each site). From 103 sites, 14 online platforms for independent adverts were selected. These were selected based on recommendations from co-researchers for being the most commonly used, or because they included the largest numbers of individual profiles, they were pre-filtered for duplicate profiles (i.e. sex workers could only post one advert/profile), they advertised individual sex worker phone numbers (as opposed to having a central number to dial like an agency), or they represented diversity among sex working population (i.e. specialist sites for men, gender fluid / transgender sex workers). From these 14 sites, we collected 11532 phone numbers from 13097 profiles. After deduplication, approximately 7746 individual profiles were identified of whom 4855 were invited to participate via sms or email or phone call. Previous indoor outreach to venues and neighbourhood walks to collect information from adverts posted in local neighbourhood identified 7 physical venues. Outreach with specialist sex worker support services identified 5 street locations, which gave 14 time-location sampling blocks.

We used time-location sampling of sex workers working on the street and targeted sampling of online profiles. We boosted slow uptake with convenience sampling (e.g. in NHS-clinics, snowball sampling), expanded recruitment of indoor sex workers to the whole of London on the basis they are more mobile than those working on the street[1] and lengthened baseline recruitment for sex workers working on the street (fig 1). The research team invited sex workers in-person, by email or phone to self-complete a structured questionnaire on a tablet or online (Open Data Kit version 1.28.4[2]) or, when requested, administered by the team. The survey was available in multiple languages spoken by sex workers in East London (English, Brazilian Portuguese, Polish and Romanian) and members of the research team were fluent in some of these languages. Three attempts to follow up participants by phone, email and street outreach to original recruitment locations. Data were collected on demographics, organisation of sex work, health and social service use, emotional and physical health, contact with police and immigration officers, violence, sexual and substance use practices. Indicators were drawn from validated measures where possible such as the Patient Health Questionnaire (PHQ)-2 and Generalised anxiety disorder (GAD)-2 screens for depression and anxiety respectively[3-5], the AUDIT-C screen measuring alcohol use[6], the Minimum European Health Module (MEHM), items from the WHO Multi-country study of violence against women (Q704-706, see

supplement table 1)[7-9] or developed to be comparable with other sex worker surveys and based on existing and emerging insights from qualitative data.

Research team safety

During all fieldwork visits research team staff were instructed to check in and check out with a research leader at the start and end of a fieldwork shift. Night fieldwork was scheduled for 6-8 hour shifts to allow a balance between safety and sufficient time to meet and recruit sex workers. Night fieldwork teams always went out in pairs, at least one member of the pair had experience of working on the street or outreach experience for services. For safety, cars were used throughout night fieldwork, but research pairs would walk up and down the streets nearby the stationed car. For night fieldwork, teams were equipped with emergency buttons similar to ones used by NHS and care workers to make home visits. These provide updated GPS coordinates and have a quick dial to emergency services in case of problems. In addition, LSHTM security made check-in calls every hour or every two hours throughout the fieldwork to update our location and ask about any issues. Interviews for night fieldwork were conducted in 24 hour restaurants or scheduled for a later day if a restaurant was not available. On rare occasions (<5) interviews were conducted within team members' cars. The research lead was always on-call in case of questions, security or safeguarding issues. Regular team briefings at the end of each fieldwork ensured any issues of safeguarding were acted on promptly should they arise as well as allowing space for research teams to talk through the interviews and any challenging topics that came up during the interview.

Biological sample collection

Voluntary self-administered Chlamydia, Gonorrhoea and HIV screening was offered in-person or by post. Alternatively, participants were asked if they consented to their last test result from participating clinics recorded as part of the research. HIV screening in-person was administered using OraSure rapid oral tests; blood prick tests were used for postal screening and positive results confirmed by Western blot. Positive results were delivered within 24 hours by SC, consultant in sexual health medicine who arranged confirmatory testing. Negative results were delivered by automated text message or within 72 hours by the project team.

1. Goldenberg SM, Chettiar J, Nguyen P, et al. Complexities of Short-Term Mobility for Sex Work and Migration among Sex Workers: Violence and Sexual Risks, Barriers to Care, and Enhanced Social and Economic Opportunities. *Journal of Urban Health* 2014;91(4):736-51. doi: 10.1007/s11524-014-9888-1
2. Hartung C, Lerer A, Anokwa Y, et al. Open data kit: tools to build information services for developing regions. Proceedings of the 4th ACM/IEEE International Conference on Information and Communication Technologies and Development. London, United Kingdom: Association for Computing Machinery, 2010:Article 18.
3. Kroenke K, Spitzer RL, Williams JB. The Patient Health Questionnaire-2: validity of a two-item depression screener. *Med Care* 2003;41(11):1284-92. doi: 10.1097/01.MLR.0000093487.78664.3C [published Online First: 2003/10/30]
4. Kroenke K, Spitzer RL, Williams JB, et al. An ultra-brief screening scale for anxiety and depression: the PHQ-4. *Psychosomatics* 2009;50(6):613-21. doi: 10.1176/appi.psy.50.6.613 [published Online First: 2009/12/10]
5. Kroenke K, Spitzer RL, Williams JB, et al. Anxiety disorders in primary care: prevalence, impairment, comorbidity, and detection. *Ann Intern Med* 2007;146(5):317-25. doi: 10.7326/0003-4819-146-5-200703060-00004 [published Online First: 2007/03/07]
6. Public Health England. Alcohol use disorders identification test consumption (AUDIT C). Available online: https://assets.publishing.service.gov.uk/government/uploads/system/uploads/attachment_data/file/684826/Alcohol_use_disorders_identification_test_for_consumption__AUDIT_C_.pdf. Alcohol use screening tests. https://www.gov.uk/government/publications/alcohol-use-screening-tests, 2017.
7. Garcia-Moreno C, Jansen HA, Ellsberg M, et al. WHO Multi-country Study on Women's Health and Domestic Violence against Women. Geneva, Switzerland: World Health Organisation 2005.
8. Schraiber LB, Latorre Mdo R, Franca I, Jr., et al. Validity of the WHO VAW study instrument for estimating gender-based violence against women. *Rev Saude Publica* 2010;44(4):658-66. doi: 10.1590/s0034-89102010000400009 [published Online First: 2010/08/03]
9. Garcia-Moreno C, Jansen HA, Ellsberg M, et al. Prevalence of intimate partner violence: findings from the WHO multi-country study on women's health and domestic violence. *Lancet* 2006;368(9543):1260-9. doi: 10.1016/s0140-6736(06)69523-8 [published Online First: 2006/10/10]
